# Collections

**Published:** 1917-04

**Authors:** 


### EDITORIAL

### Collections.

During Newspaper Week at the School of Journalism of the University of Texas a successful editor and publisher, in emphasizing the importance of making prompt collections, stated that he would not retain the services of a physician who would not send his bills regularly and promptly. He gave as a reason for this that he could not have much confidence in the ability of a man who was so slipshod in his methods as to neglect his collections, and further that he thought that the physician who fails to render bills for his services does not esteem those services as worth much.

Perhaps that was an extreme statement, but business men do have little patience with the fellow who doesn't use business methods, and they are inclined on that account to cease having anything to do with him. Most men like to pay their bills promptly; at least they like to know what they are so that they may pay them when they are able to do so.

Most disagreements as to accounts are caused by unnecessary delays in their presentation. An account long delayed is looked upon with suspicion by the party receiving it, and is likely to be considered too large.

The time when the patient feels most kindly toward the physician is just after the services have been rendered and when the ministrations are so fresh that they are appreciated. The psychology of business teaches that the best time to collect for services is while there is an appreciative remembrance of those services.

Physicians more than any other class are proverbially slow in making their collections, and consequently are often forced to be slow in paying their debts, thus losing at both ends of business and suffering in popular esteem. They are to blame for creating the idea that the time to pay them is after all other obligations are paid, and then only in case there is anything left over with which to pay.

Sentiment should not be allowed to interfere with the business side of the practice of medicine, and the physician should remember that, however lax he may be in making collections, those whom he owes expect him to be prompt in settling with them.

